# Implementation of Ultrasonic Sensing for High Resolution Measurement of Binary Gas Mixture Fractions

**DOI:** 10.3390/s140611260

**Published:** 2014-06-24

**Authors:** Richard Bates, Michele Battistin, Stephane Berry, Alexander Bitadze, Pierre Bonneau, Nicolas Bousson, George Boyd, Gennaro Bozza, Olivier Crespo-Lopez, Enrico Da Riva, Cyril Degeorge, Cecile Deterre, Beniamino DiGirolamo, Martin Doubek, Gilles Favre, Jan Godlewski, Gregory Hallewell, Ahmed Hasib, Sergey Katunin, Nicolas Langevin, Didier Lombard, Michel Mathieu, Stephen McMahon, Koichi Nagai, Benjamin Pearson, David Robinson, Cecilia Rossi, Alexandre Rozanov, Michael Strauss, Michal Vitek, Vaclav Vacek, Lukasz Zwalinski

**Affiliations:** 1 SUPA School of Physics and Astronomy, University of Glasgow, G627QB, UK; E-Mails: richard.bates@glasgow.ac.uk (R.B.); Alexander.Bitadze@cern.ch (A.B.); 2 CERN, 1211 Geneva 23, Switzerland; E-Mails: Michele.Battistin@cern.ch (M.B.); Stephane.Berry@cern.ch (S.B.); Pierre.Bonneau@cern.ch (P.B.); gennaro.bozza@cern.ch (G.B.); Olivier.Crespo-Lopez@cern.ch (O.C.-L.); enrico.da.riva@cern.ch (E.D.R.); Beniamino.Di.Girolamo@cern.ch (B.D.); Gilles.Favre@cern.ch (G.F.); Jan.Godlewski@cern.ch (J.G.); Didier.Lombard@cern.ch (D.L.); lukasz.zwalinski@cern.ch (L.Z.); 3 Centre de Physique des Particules de Marseille, 163 Avenue de Luminy, 13288 Marseille, France; E-Mails: nicolas.bousson@cern.ch (N.B.); gregh@cppm.in2p3.fr (G.H.); nicolas.langevin.nl@gmail.com (N.L.); mathieu@cppm.in2p3.fr (M.M.); mathieu@cppm.in2p3.fr (A.R.); 4 Department of Physics & Astronomy, University of Oklahoma, Norman, OK 73019, USA; E-Mails: a.hasib@cern.ch (A.H.); benpearson@ou.edu (B.P.); strauss@nhn.ou.edu (M.S.); 5 Department of Physics, Indiana University, Bloomington, IN 47405 7015, USA; E-Mail: Cyril.Degeorge@cern.ch; 6 Deutsches Elektronen-Synchrotron, Notkestraβe 85, D-22607 Hamburg, Germany; E-Mail: cecile.deterre@desy.de; 7 Czech Technical University, Technická 4, 16607, Praha Czech Republic; E-Mails: Martin.Doubek@cern.ch (M.D.); miguel.vitek@gmail.com (M.V.); Vaclav.Vacek@cern.ch (V.V.); 8 B.P. Konstantinov Petersburg Nuclear Physics Institute (PNPI), Orlova Roscha, RU-188300, Russia; E-Mail: Sergey.Katunin@cern.ch; 9 STFC Rutherford Appelton Laboratory—Harwell Oxford, Didcot, OX11 OQX, UK; E-Mail: steve.mcmahon@stfc.ac.uk; 10 Department of Physics, Oxford University, Oxford OX1 3RH, UK; E-Mail: Koichi.Nagai@cern.ch; 11 Department of Physics and Astronomy, University of Cambridge, Cambridge, CB3 0HE, UK; E-Mail: dave.robinson@cern.ch; 12 Academy of Sciences of the Czech Republic, 11000 Praha, Czech Republic; E-Mail: cecilia.rossi@cern.ch

**Keywords:** ultrasonic, binary gas analysis, leak detection

## Abstract

We describe an ultrasonic instrument for continuous real-time analysis of the fractional mixture of a binary gas system. The instrument is particularly well suited to measurement of leaks of a high molecular weight gas into a system that is nominally composed of a single gas. Sensitivity < 5 × 10^−5^ is demonstrated to leaks of octaflouropropane (C_3_F_8_) coolant into nitrogen during a long duration (18 month) continuous study. The sensitivity of the described measurement system is shown to depend on the difference in molecular masses of the two gases in the mixture. The impact of temperature and pressure variances on the accuracy of the measurement is analysed. Practical considerations for the implementation and deployment of long term, *in situ* ultrasonic leak detection systems are also described. Although development of the described systems was motivated by the requirements of an evaporative fluorocarbon cooling system, the instrument is applicable to the detection of leaks of many other gases and to processes requiring continuous knowledge of particular binary gas mixture fractions.

## 1. Introduction

**Figure 1.**
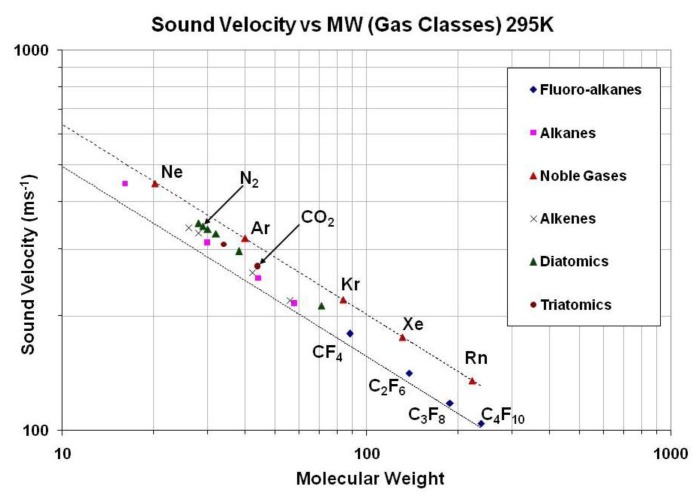
The speed of sound in a gas mixture can be very sensitive to the ratio of the two components, depending on the difference in their molecular masses.

Fractional measurement of binary gas mixtures with ultrasonic pulses has been in use for some decades in High Energy Physics (HEP) experiments; see for example [1]. These experiments use binary gas mixtures that must be accurately monitored and controlled in order to optimize the experimental data. Monitoring in these experiments is achieved by gas mixture analyzers operating on the principle that the speed of sound in binary gas mixtures of differing molecular masses is sensitive to the ratio of the molar fraction of the mixture (Figure 1).

Recently, we have expanded the application of ultrasonic gas analysis to the silicon tracker of the ATLAS experiment (A Toroidal LHC ApparatuS) at the CERN Large Hadron Collider (LHC) for *in situ* leak detection [2].

The silicon detectors of the ATLAS Inner Detector are subdivided into the ATLAS Pixel Detector, using pixelated silicon sensors [3] and the SCT Detector (Semi-Conductor Tracker), constructed of silicon micro-strip sensors. They share a common evaporative cooling system using that uses the coolant octafluoropropane (C_3_F_8_), a saturated (C_n_F_(2_*_n_*_+2)_) fluorocarbon with convenient thermodynamics. C_3_F_8_ also features radiation resistance, non-toxicity, non-flammability and non-conductivity [4]. The coolant is delivered via capillaries (which cannot be allowed to clog) in 204 independently controlled cooling loops and provides local cooling to an average of approximately −7 °C for the silicon sensors and their associated electronics, with a total design capacity of 60 kW of heat removal [5]. In its present form, the cooling uses a conventional compression → condensation → detent cycle but the oil-free compressor plant is currently being replaced with a passively pumped thermosiphon [6]. The thermosiphon system uses the 92 m height difference between the surface and the ATLAS underground experimental caverns, in combination with the thermodynamic cycle, to drive the coolant circulation. In this design, the necessary liquid delivery pressure will be provided by the hydrostatic column of 92 m of C_3_F_8_ liquid, while the C_3_F_8_ vapor will climb to the surface condenser, the lowest pressure element in the system. The headspace above the coolant in the liquid reserve vessel is also at sub-atmospheric pressure and will need to be monitored for the ingress of air. A passive ultrasonic leak detection system is ideal in this application to respect the purity of the gas circulating in the system.

The aforementioned silicon detector systems had been suspected of leaking small amounts of coolant into each of their segregated, nitrogen-flushed environments from the time of their installation in 2006–2007. The possible leaks are of concern because the high ionizing radiation fluence of the ATLAS inner detector environment could dissociate C_3_F_8_ coolant molecules, possibly leading to the creation of hydrofluoric acid (HF) if it should happen that a detector gas volume were to be accidentally exposed to humidity during detector operation. This could lead to corrosion damage to the (millions of) exposed Al wire bonds (wire bonds are very thin, typ. 1 mil, for short connections between circuit components, most usually between an integrated circuit and an interconnect, such as a printed circuit board or standardized package.) used in the detectors, rendering the associated detector modules with limited or no functionality, creating (a) blind spot(s) in the Inner Detector of the ATLAS experiment. Because the detector volumes of the Pixel and SCT sub-detectors are separately flushed with N_2_ to prevent condensation, the possibility exists to extract and analyze each volume exhaust for coolant leaks.

In this paper we describe how the application of existing detector technology for ultrasonic fractional binary gas analysis in new hardware and control systems can provide sensitive, *in situ*, passive leak detection. We also describe design options that give the ultrasonic sensing system additional functionality.

## 2. Theory, Design and Implementation of Ultrasonic Leak Detection in Binary Gas Systems

The principle of operation and implementation of the ultrasonic leak detection system is described in the following three subsections.

### 2.1. Principle of Operation

The principle for leak detection using ultrasonic acoustic waves is based on the general equation for sound velocity in a gas:

(1) $$c=\sqrt{\frac{\gamma \text{RT}}{M}}$$

where *γ* is the adiabatic index of the gas, *R* is the molar gas constant (8.3145 J·mol^−1^·K^−1^), *T* is the absolute temperature in degrees Kelvin and *M* is the molar mass in kg·mol^−1^. The value of *γ* is given by the ratio of the molar specific heat at constant pressure (*Cp*) to that at constant volume (*Cv*):

(2) $$\gamma =\frac{Cp}{Cv}$$

When dealing with a gas mixture, one calculates the sum of the specific heat components in proportion to the molar fraction, *w*_i_, of each:

(3) $$\gamma_{m}=\frac{C_{\text{pm}}}{C_{\text{vm}}}=\frac{\sum_{i}w_{i}Cp_{i}}{\sum_{i}w_{i}Cv_{i}}$$

By definition, the molar mass of the mix is given by:

(4) $$M=\sum_{i}w_{i}M_{i}$$

So Equation (1) becomes:

(5) $$c=\sqrt{\frac{\frac{\sum_{i}w_{i}Cp_{i}}{\sum_{i}w_{i}Cv_{i}}\text{RT}}{\sum_{i}w_{i}M_{i}}}$$

For each mixture component, the molar specific heat at constant pressure and that at constant volume are related by:

(6) $$Cp_{i}-Cv_{i}=R$$

The precision of mixture determination, *∂*(mix), at any concentration of the two components is given by:

(7) $$\partial (\text{mix})=\frac{\partial c}{m}$$

where *m* is the local slope of the sound velocity-concentration curve and *∂c* is the uncertainty in the sound velocity measurement.

In a system composed of N_2_ and C_3_F_8_, for example, the mixture ratio can be expressed as *w_N_*_2_ = (1 − *w_C_*_3_*_F_*_8_), reducing Equation (5) to a function of *w_C_*_3_*_F_*_8_, *Cp_C_*_3_*_F_*_8_ and *Cp_N_*_2_. The two molar specific heats can be computed using NIST REFPROP [7] or with PC-SAFT [8] over a range of temperature and pressure exceeding the conditions in the analyzer tube(s) and then stored in a database (look-up table). Therefore, we only need measure the speed of sound in the mixture along with the temperature profile along the path of the gas (for improved accuracy) and the gas pressure, in order to calculate *w_C_*_3_*_F_*_8_/*w_N_*_2_.

### 2.2. Implementation

**Figure 2.**
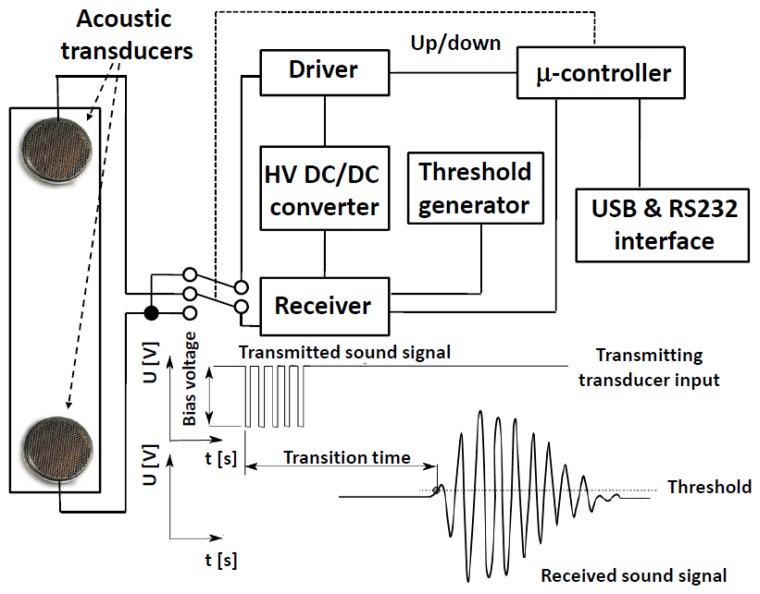
An illustration of the ultrasonic signal transit time measurement and the electronic readout block diagram is shown for a design that allows the transducer roles to be swapped for more precise calibration and for simultaneous gas velocity measurement.

**Figure 3.**
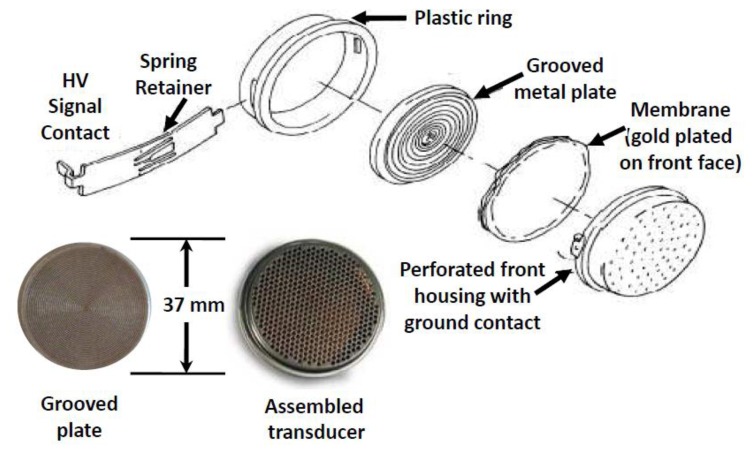
Senscomp model 600 capacitive ultrasonic transducer originally designed by Polaroid^®^ for autofocus cameras.

In our system designs, ultrasonic bursts are propagated in a sealed tube designed to provide a smooth flowing gas region between two transducers (Figure 2). The transducers were designed by Polaroid^®^ and are now sold by Senscomp (Figure 3) as series 600 [9]. The transducer is comprised of a thin, Au plated Mylar^®^ foil stretched over a spirally grooved conductive disk. The foil is held at ground potential and the disk is biased between 100 Vdc and 360 Vdc. A 50 kHz pulse train modulates the transducer bias voltage, exciting the diaphragm to transmit an acoustic wave in response to the fluctuating electric field. As a sensor, the transducer collects an alternating charge across the foil—disk capacitor that is proportional to the power of the signal impinging on the foil diaphragm. A 50 Vdc bias is adequate in receive mode to maximize the signal to noise ratio of the transducer in the gases we have studied. An appealing feature of this sensor is that its construction maintains pressure equilibrium across the diaphragm, making the pressure in the gas system irrelevant to transducer operation. Its suitability for use with corrosive gases has not been examined but these sensors have displayed long lifetimes in the applications reported here (none are known to have failed).

Most of the electronics needed to drive the transducers, create gated pulses and measure the time of flight of pulses between the transmit and receive transducers can be implemented in a micro-controller. The Microchip^®^ dsPIC 16 bit microcontroller has been chosen for the present ATLAS design. It provides a 40 MHz timer for measurement of the sound transit time through the gas or gas mixture that is started when the first ultrasonic pulse is sent. The microcontroller also controls the gain of the analog amplifier and generates an analog comparison voltage for the discriminator, whose output signal triggers the 40 MHz timer to halt. The discriminator follows the analog amplifier to boost the received transducer signal and shape it. It is also possible to design the system to take alternating measurements in opposite directions, relative to the gas stream, so that the analog amplifier, cabling and discriminator delays are eliminated from the time of flight measurements. This technique also allows passive gas velocity measurement in the same instrument that is used for leak detection [10].

It is possible to use the same microcontroller to store the sound velocity-composition lookup tables and locally calculate the mixture ratio. However, ATLAS is structured around a control and monitoring system that makes it more practical to perform lookup and other functions in a supervisory computer that can serve several analyzer tubes. Communications protocols are determined by the specific application. In ATLAS, we will convert to MODBUS over TCP/IP but have also used and will continue to use RS-232C and USB for local digital communication and 4–20 mA current loops for hard-wired control functions.

The gas mixture and flow rate are continuously calculated using Supervisory Control and Data Acquisition (SCADA) software implemented in Siemens WinCC^®^ (formerly known as PVSS II [11]), running on the supervisory computer. Sound velocity-concentration lookup tables are used to accelerate execution speed and are stored on the same computer. These tables may be created from prior measurements in calibration mixtures, theoretical thermodynamic calculations, published tables or a combination of these sources.

After completion of the full ultrasonic analysis network in 2014, a central computer will be used to supervise the four leak check systems described in this paper, as well as another ultrasound system for passively measuring the gas flow velocity in the thermosiphon vapor returning to the surface condenser, which operates at sub-atmospheric pressure [12].

The acoustic wave has a very low angle of dispersion at 50 kHz because it is driven from a diaphragm that is much larger (typically by a factor of 10) than the acoustic wavelength of the probe signal. The important features of a tube for ultrasonic gas analysis include mounts for the transducers that keep the transducer diaphragms aligned parallel to each other in the gas stream. Misalignment can lead to increased errors due to reflections from the tube walls and/or attenuation of the signal. Since the wavelength of the ultrasonic signals is very short, the choice of length for the measurement tube is driven by other considerations, including mounting space and the precision of the timer(s) used to measure the time of flight of the probe signal. Provision should also be made to seal the transducer wiring ports, including the wiring for any temperature and pressure sensors that might be included inside the tube for improved accuracy. Care should be taken to avoid creating acoustic resonators or vortices in the analyzer tube design that could interfere with the measurement. The tube should be installed in a system line that is free of liquid, aerosols, risk of condensation and other contaminants.

**Figure 4.**
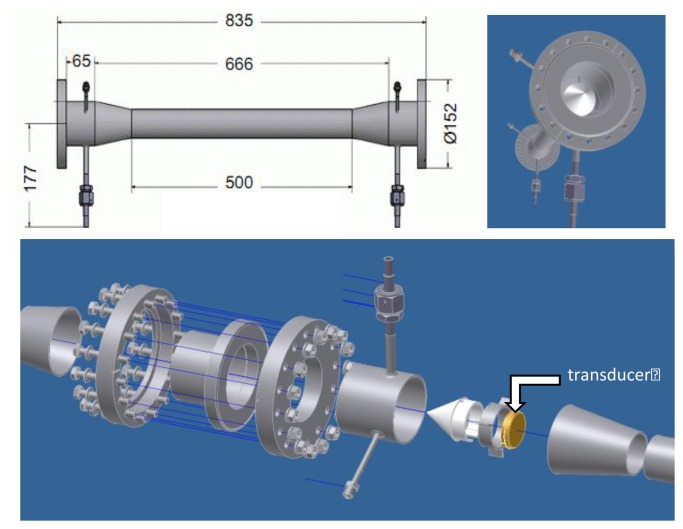
The schematics show the implementation of a stainless steel analyzer tube, with the transducer highlighted in gold in bottom detail. The central tube inner diameter is pinched to be the same as the ultrasonic transducers. This version allows volume flow measurement to ∼230 L min^−1^, in addition to the measuring the gas mixture ratio.

A tube design allowing simultaneous binary gas analysis and velocity measurement (flowmetry), has been modeled using computational fluid dynamics [13]. One such tube design for axial flowmetry is shown in Figure 4. Note the cones that reduce chaotic fluid flow and vortices as gas flows around the transducers. Although stainless steel is chosen for the analyzer tube construction in our applications, other materials may be more suitable in others. The design should anticipate pipe stems, valves and manifolds to ease calibration, servicing and replacement of the sensing tube and/or elements within it.

As mentioned in the introduction, it is possible to monitor the N_2_ environmental volumes of the ATLAS inner detector for C_3_F_8_ leaks by remotely sampling their atmospheres. Dry N_2_ is constantly flowing to purge the detector volumes of any humidity, and gas can be aspirated from different locations through seven 8 mm diameter exhaust tubes. These tubes traverse a path of 150 m length from the ATLAS Inner Detector to three ultrasonic analyzers installed in an underground service cavern, a safe distance from the high radiation environment of the experimental cavern. One of these tubes is dedicated to sampling the environmental volume of the ATLAS Pixel Detector and two others sample the ATLAS SCT Detector. Another tube is routed to a soon-to-be-installed new silicon pixel detector, known as the “Insertable B Layer” (IBL), equipped with its own separately purged N_2_ environmental volume. Since the IBL will be evaporatively cooled using a new CO_2_ based system, we are extending the analysis capability of the ultrasonic instrument to include CO_2_ leaks into N_2_ [14].

**Figure 5.**
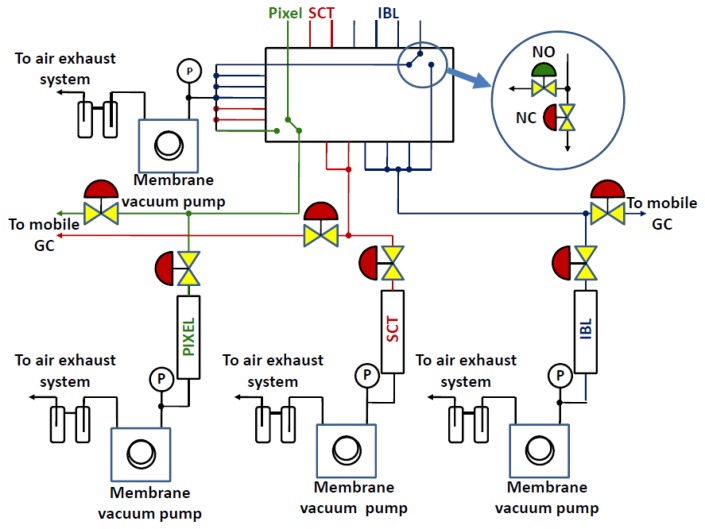
Schematic of the ATLAS inner detector leak detection system. Two ultrasonic analyzer tubes target detection of C_3_F_8_ leaks into the N_2_ environments surrounding the Pixel and SCT detectors. A third tube targets CO_2_ leaks into the IBL gas volume. A portable gas chromatograph (GC) can be connected as a calibration check or if more than one contaminating gas is suspected [15].

Figure 5 illustrates the gas sampling system in which gas is pumped through three ultrasonic gas analysis tubes using low volume flow (∼100 cm^3^·min^−1^) diaphragm pumps. A pneumatic valve selection panel located in the service cavern allows the operator to remotely choose (or program) which detector volumes to analyze.

**Figure 6.**
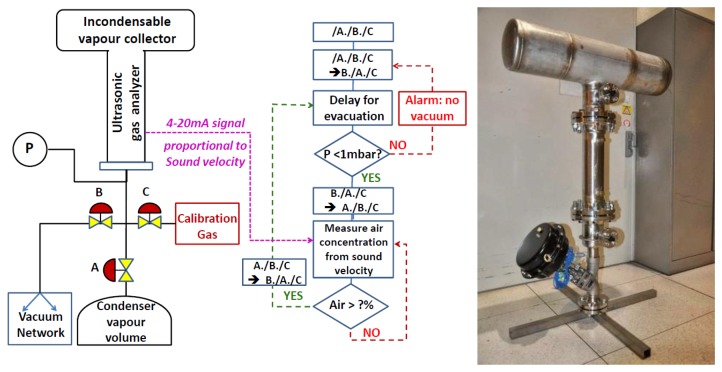
The schematic and logic flow of the leak detection system of the condenser headspace of the ATLAS inner detector thermosiphon cooling system is shown on the left. A valve system is included which allows for filling the analyzer tube with a calibration gas and purging the tube with a vacuum pump. The completed analyzer and valve tree is shown on the right, prior to installation.

In the thermosiphon condenser application, a vertical tube system is required that can be isolated from the condenser for maintenance and calibration. Fortunately this is simplified by being a part of the head-space purging system of the coolant reserve tank, which can be isolated from the rest of the tank (Figure 6). The condenser is located outdoors, far from rack space for computers and other equipment, so for this system, the electronics have been implemented within a DIN rail compliant weatherproof equipment box. Communication will be by MODBUS over TCP/IP to the supervisory computer, which is the same one used for leak detection, located in an equipment rack with the three analyzer tubes mentioned previously.

### 2.3. Calibration

Precision measurement of the mixture is achieved by accurately determining the distance between the transducers, *i.e.*, calibrating the analyzer tube length. The sound transit time can be calibrated by filling the analyzer tube with a pure gas. The best candidate gases are those with well-known sound velocity dependence on temperature and pressure, including xenon, whose sound velocity (175.5 ms^−1^ at 20 °C) is the closest to that of the fluorocarbon gases analyzed in ATLAS and for which the thermo-physical behavior is that of an ideal gas. Sufficient precision is often possible using nitrogen or argon, which are considerably cheaper and more widely available. The average uncertainty in transducer inter-distance measured in this way is ±0.1 mm. Of obvious importance to calibration is control of the temperature and pressure or at least, their accurate measurement (Equations (1)–(6)). For even tighter calibration, the design can include a thermal jacket around the analyzer tube. In the instruments we have deployed, the combined effects of uncertainties in the transducer spacing and the measured temperature (<±0.2 °C) and pressure (<±4 mbar) in the tube result in an uncertainty in sound velocity measurement of <±0.05 ms^–1^. In combination with the stored sound velocity-concentration look up tables, this results in an uncertainty of the gas mixture determination, calculated with Equation (7), depending on the molecular weight difference of the two components, as discussed in the following sections.

## 3. Results and Discussion

### 3.1. Examples of Velocity-Concentration Look-UP Tables

**Figure 7.**
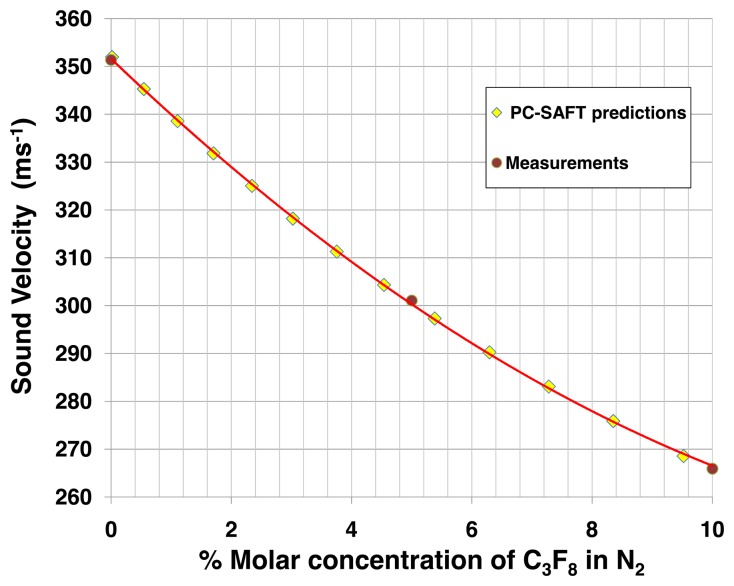
Comparison of sound velocity measurements and PC-SAFT predictions in C_3_F_8_/N_2_ mixtures at 1bar abs and 25 °C up to 10% molar concentration.

**Figure 8.**
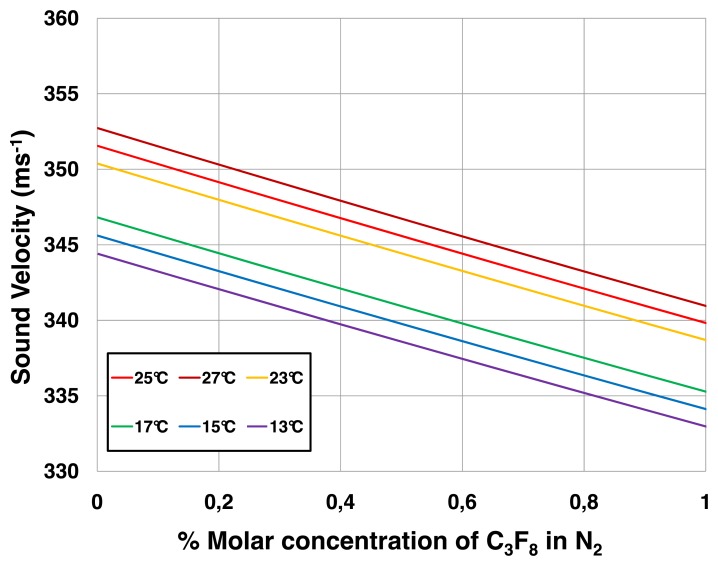
Sound velocity calculated according to the formalism of Equations (1)–(6) for C_3_F_8_/N_2_ molar concentrations in the range (0%–1% C_3_F_8_) at 1bar_abs_ and several temperatures.

Figures 7 and 8 illustrate look up tables applicable to leak detection of C_3_F_8_ into N_2_. Figure 7 compares measured sound velocities in molar concentrations of up to 10% C_3_F_8_ in N_2_ with direct sound velocity predictions made using the PC-SAFT equations of state [8]. The data of Figure 7 are abstracted from measurements and predictions made over the full concentration range from pure C_3_F_8_ to pure N_2_. The PC-SAFT approach was used, as NIST-REFPROP is not presently configured for direct sound velocity calculations for mixtures of saturated fluorocarbons with N_2_. In an alternative approach illustrated in Figure 8, sound velocity may be determined using the formalism of Equations (1)–(6) using C_p_ and C_v_ data for the two component gases separately calculated using NIST-REFPROP over a range of temperature and pressure encompassing the process gas conditions.

The large difference in molecular weight between C_3_F_8_ and N_2_ (respectively 188 and 28 units) affords very high sensitivity to variations in the C_3_F_8_ leak concentration. For example in the (0%-1%) molar concentration (MC) range of most interest in leak detection of a heavy vapor into a light carrier, the slope of the sound velocity-concentration curve is −12.2 ms^−1^.(%MC)^−1^. This slope, taken in combination with the sound velocity measurement error of ±0.05 ms^−1^ using Equation (7), results in a mixture resolution of ±4 × 10^−5^.

**Figure 9.**
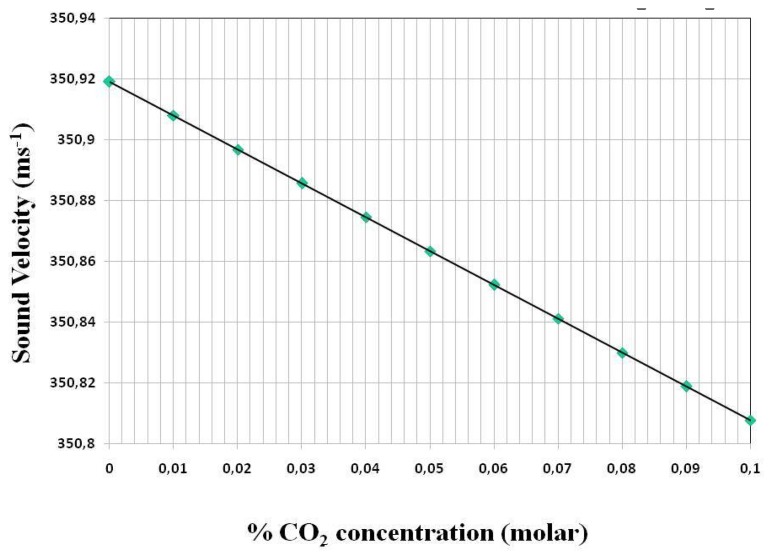
Sound velocity calculated according to the formalism of Equations (1)–(6) for CO_2_/N_2_ mixtures in the molar concentration range (0%–0.1% CO_2_) at 1bar_abs_ and a tube operating temperature of 21.8 °C.

Figure 9 illustrates the variation of sound velocity with the concentration of CO_2_ leaking into a N_2_ atmosphere, as in the case of the environmental volume of the new ATLAS IBL. Here the smaller difference in molecular weight between CO_2_ and N_2_ (respectively 44 and 28 units) results in a shallower slope to the sound velocity-concentration curve. For example in the (0%–0.1%) molar range of most interest in leak detection the slope of the sound velocity-concentration curve is −1.12 ms^−1^(%MC)^−1^. This slope, taken in combination with the sound velocity measurement error of ±0.05 m/s using Equation (7), results in a mixture resolution of ±4.4 × 10^−4^.

**Figure 10.**
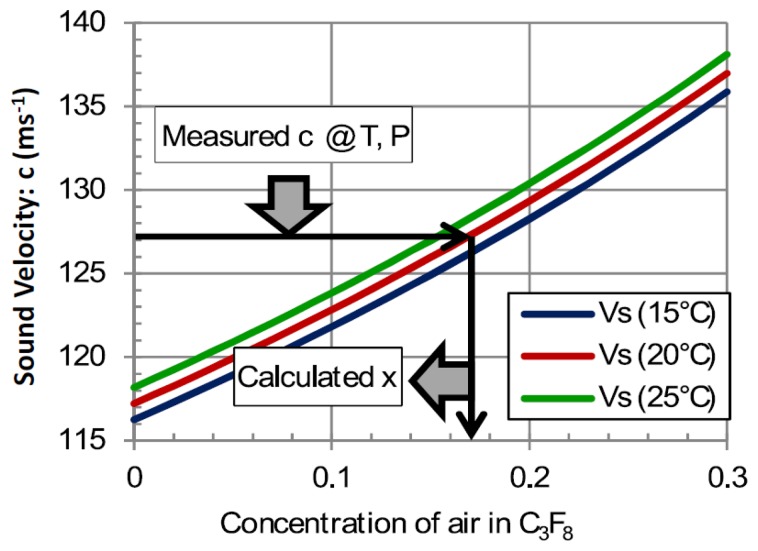
The sound velocity is calculated according to the formalism of Equations (1)–(6) for C_3_F_8_/air mixtures in the molar concentration range (0%–30% air) at 1 bar_abs_ at three tube operating temperatures [16].

Figure 10 illustrates the variation of sound velocity with the concentration of air leaking into the C_3_F_8_ atmosphere in the headspace of the thermosiphon condenser. Although the difference in molecular weight between C_3_F_8_ and air (respectively 188 and 29 units) is still very large, this instrument operates at the opposite end of the spectrum to the example shown in Figures 7 and 8.

In this case, a light contaminant leaks into a heavy carrier. The slope for the corresponding end of the velocity-concentration curve is shallower, e.g., in the (0%–10%) molar range of most interest in this application the slope of the sound velocity-concentration curve is 0.53 ms^−1^ (%MC)^−1^. This slope, taken in combination with the sound velocity measurement error of ±0.05 ms^−1^ and using Equation (7), results in a mixture resolution of ±9.4 × 10^−4^.

### 3.2. Results from Operation of the Devices

**Figure 11.**
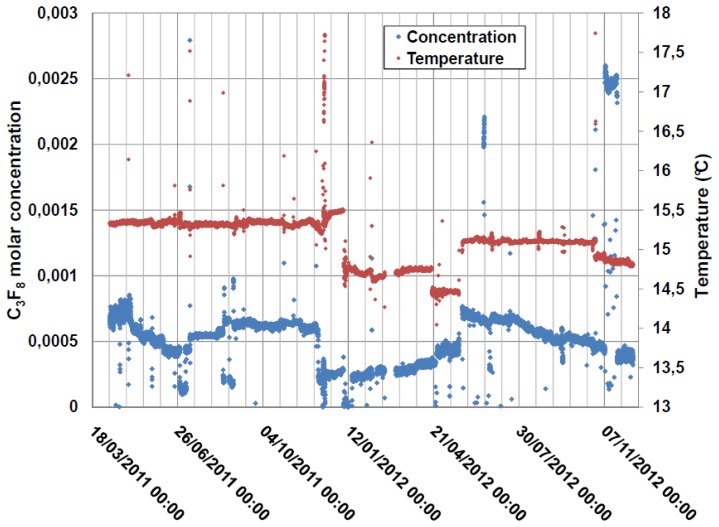
This plot of the long duration (>18 month) log of C_3_F_8_ leak contamination in the N_2_ environmental gas surrounding the ATLAS Pixel Detector confirms suspected leaks and establishes the leak rate into the detector volume [12].

The N_2_ atmosphere surrounding the ATLAS pixel detector was continuously monitored from March 2011 until the programmed 18-month shutdown of the CERN LHC in February 2013. Gas was aspirated from the pixel detector volume at around 100 mL/min with the analyzer tube operating at a pressure of (985 ± 2) mbar_abs_ and a typical operating temperature of 15.2 °C. The C_3_F_8_ concentration as a function of time is shown in Figure 11. The electronics had several downtimes of a few weeks, which caused the discontinuity in the distribution. The short periods of drops to zero C_3_F_8_ concentration correspond to periodic baseline checks with pure N_2_. Longer period fluctuations are due to variations in the nitrogen purge rate through the detector environmental volume. The peak in apparent C_3_F_8_ concentration seen in November 2012 was due to the simultaneous aspiration of air into the tube through an improperly tightened gas fitting, the apparent C_3_F_8_ concentration being based on the increased sound transit time and correspondingly reduced sound velocity in the tube. A reduction in sound velocity of 0.86 ms^−1^ from that of pure nitrogen is typically observed when the full Pixel Detector cooling system of 88 independent circuits is fully operating. From the ∼12.27 ms^−1^·(%MC)^−1^ average gradient of the sound velocity-concentration curve for C_3_F_8_ concentrations in the range 0%–0.5% (Figures 7 and 8) this sound velocity difference indicates, via Equation (7), a C_3_F_8_ ingress of 0.07% (Figure 11).

Both the pressure and the temperature of the gas in the tube are continually measured electronically, and a full interpolation is made between points of the created (c; P; T; C_3_F_8_ concentration) database.

**Figure 12.**
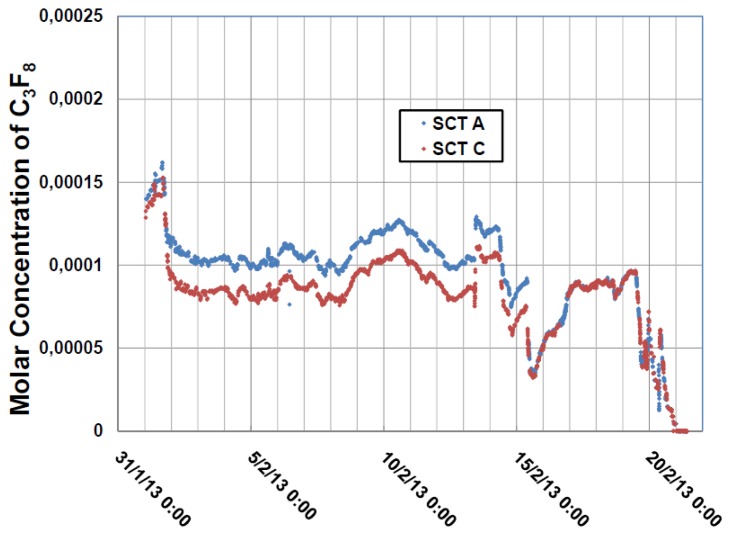
The variance in concentration of C_3_F_8_ in the SCT gas enclosure as a function of time (between 1st and 21st February) is shown for the two zones analyzed, designated SCTA and SCTC [12].

Gas was aspirated from two different zones of the SCT detector N_2_ envelope into an analysis tube that was operated for three weeks in February 2013 with the latest version of the electronics, until the LHC shutdown. Figure 12 illustrates the concentrations of C_3_F_8_ during that period. Through the use of the automated sampling system of Figure 5, gas was alternately sampled for a period of one hour from each zone. The variance between the two zones (upstream and downstream into the SCT environmental volume with respect to the direction of dry nitrogen gas injection) was seen to diminish after 15 February when the purge rate was increased.

ATLAS commenced a long (2013–2014) maintenance and upgrade shutdown, ceasing operations in mid-February 2013. On the 20th of that month the cooling circuits of the ATLAS silicon detectors were progressively turned off, starting with those of the SCT and followed by those of the Pixel detectors. The cooling shutdown and elimination of C_3_F_8_ from the cooling circuits was complete by 00:00 on February 21. Before the start of this process, an N_2_ baseline was taken for the instrument analyzing the pixel detector environmental gas.

**Figure 13.**
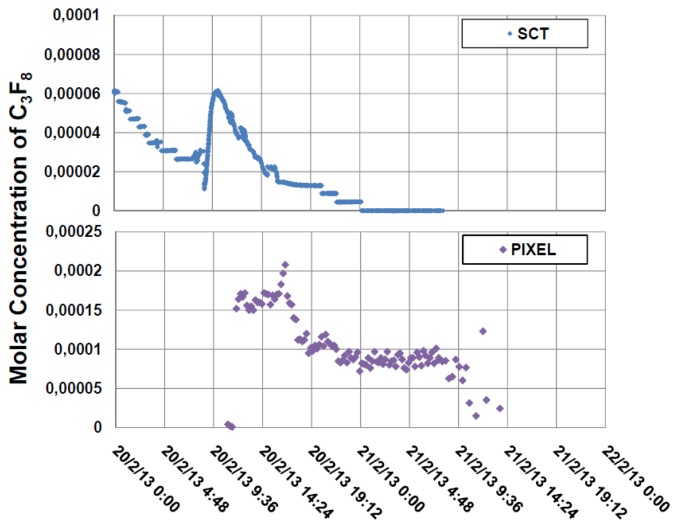
The variance in concentration of C_3_F_8_ is shown for the Pixel (**right**) and SCT (**left**) gas enclosures during the shutdown of the cooling systems on 20th February 2013, the latter of which was regularly alternated between the SCTA and SCTC zones during the shutdown [12].

The residual C_3_F_8_ contamination in both zones of the SCT N_2_ envelope was seen to drop to zero within around 15 h of the commencement of the SCT cooling shutdown. The Pixel Detector cooling shutdown protocol was longer due to known leaks in some of the cooling circuits. Residual C_3_F_8_ thus continued to be aspirated from the pixel detector nitrogen envelope over a longer period. The largest drops in the C_3_F_8_ concentration were seen following the shutdown of the 3rd pixel barrel layer, which began at 17:00 on 20 February 2013 (Figure 13).

## 4. Conclusions and Outlook

We have described an ultrasonic instrument for continuous real-time binary gas composition measurement, through the combination of sound velocity, temperature and pressure measurements. The instrument is particularly well suited to measure leaks of a high molecular weight gas into a light carrier. A sensitivity of <5 × 10^−5^ has been demonstrated to leaks of octoflouropropane (C_3_F_8_) coolant into nitrogen during a long duration (18 month) continuous study. Sensitivity studies suggest that mixture resolutions of <9.5 × 10^−4^ and <4.5 × 10^−4^ respectively will be possible in C_3_F_8_ /air and CO_2_/N_2_ mixtures of interest to the ATLAS inner detector cooling project. Although this development was motivated by the requirements of an evaporative cooling system, the instrument is applicable to the detection of leaks of many other refrigerants, and to processes requiring continuous knowledge of binary gas composition.

The Pixel and SCT volumes continue to be monitored for leaks as part of a large system that monitors the health of the ATLAS detectors. The accuracy of passive, ultrasonic leak detection has already proven its usefulness in understanding the coolant leaks in the ATLAS silicon detectors.

Operation of the condenser headspace monitoring system we have described will begin during commissioning of the new thermosiphon cooling system for the ATLAS silicon detectors during 2014. Installation has begun for the plumbing necessary to instrument the new IBL Detector volume for monitoring. Since CO_2_ is known to be highly absorbent of ultrasonic and even high audio frequencies, a new R&D program has begun to explore the severity of attenuation at high CO_2_ concentrations. Preliminary studies suggest that 50 kHz signals can be detected in our present instrument after passage through 50 cm of gas at molar CO_2_ concentrations up to 15%. We therefore also plan to investigate the use of audio frequency transducers in a new system being built at the University of Oklahoma. The results obtained will allow optimization of the tube geometry and choice of transducers in the ATLAS IBL application.

Additional uses for speed of sound measurements in parallel with the work described here include gas flowmetry and analysis of C_2_F_6_/C_3_F_8_ coolant blends for optimized thermodynamics [2].

The technology described in this work could also find application in other sectors where passive, long term, *in situ* leak detection (or fractional gas mixture analysis) is required. These include Metal Organic Chemical Vapor Deposition (MOCVD) semiconductor manufacturing, anesthesiology and hydrocarbon fuel combustion management.
